# Microglial NFAT5 aggravates neuroinflammation via mediating NLRP6 inflammasome in experimental ischemic stroke

**DOI:** 10.1016/j.gendis.2025.101614

**Published:** 2025-04-01

**Authors:** Hui Gan, Mi Zhang, Yuhao Duan, Ailiyaer Palahati, Qi He, Junyi Tan, Yong Li, Xuan Zhai, Jing Zhao

**Affiliations:** a Department of Neurosurgery Children’s Hospital of Chongqing Medical University, National Clinical Research Center for Child Health and Disorders, Ministry of Education Key Laboratory of Child Development and Disorders, Chongqing Key Laboratory of Child Neurodevelopment and Cognitive Disorders, Chongqing 400010, China; b Center for Neuroscience Research, School of Basic Medicine, Chongqing Medical University, Chongqing 400016, China; c Key Laboratory of Major Brain Disease and Aging Research (Ministry of Education), Chongqing Medical University, Chongqing 400016, China; d Department of Biochemistry and Molecular Cell Biology, Shanghai Jiao Tong University School of Medicine, Shanghai 200025, China

**Keywords:** Ischemic stroke, Microglia, Neuroinflammation, NFAT5, NLRP6 inflammasome

## Abstract

Microglial activation triggers the inflammatory cascade and exacerbates brain injury following ischemic stroke. Middle cerebral artery occlusion (MCAO) modeling increased the expression of nuclear factor of activated T cells 5 (NFAT5) in microglia. However, the role of microglial NFAT5 in ischemic stroke remains unclear. Here, our findings indicated that microglial NFAT5 knockdown reduced the expression of pro-inflammatory factors, microglial activation, and neutrophil infiltration, ultimately ameliorating cerebral infarction and neurological deficits in mice following MCAO. Additionally, we treated hippocampal neuronal cells (HT22) with a conditioned culture medium from a microglia cell line (BV2) to simulate microglia-induced neuronal injury *in vitro*. We observed that NFAT5 knockdown attenuated the expression of pro-inflammatory factors in BV2 cells and reduced apoptosis in HT22 cells. Previously, our published work reported that the NOD-like receptor pyrin domain-containing 6 (NLRP6) inflammasome contributed to inflammatory injury after MCAO. In this study, we discovered that NFAT5 promoted the transcriptional activity of the *Nlrp6* promoter through its −1527 bp to −1518 bp element. Notably, our results also demonstrated that NFAT5 regulated the stability of NLRP6 mRNA via the 5′UTR of *Nlrp6*. Thus, our findings reveal the pivotal role and partial mechanism of microglial NFAT5 in neuroinflammation following ischemic stroke.

## Introduction

Stroke has emerged as the predominant cause of mortality among adults in China, as indicated by recent epidemiological evidence.^1^ Ischemic stroke, which accounts for approximately 80%–85% of all stroke cases, is the most prevalent form of stroke. For reperfusion, intravenous tissue plasminogen activator and endovascular thrombectomy are currently the two primary therapies for acute cerebral ischemia.^2^ Nevertheless, reperfusion to ischemic brain tissue may exacerbate cerebral ischemia-reperfusion injury.^3^ Cumulative studies have concluded that neuroinflammation plays a pivotal role after ischemic stroke.^4^ Therefore, it is imperative to investigate the underlying mechanisms of neuroinflammation after ischemic stroke.

Microglia, the brain’s resident immune cells, remain in a quiescent state under normal physiological conditions.^5^ However, in response to brain injury, microglia rapidly transition to an activated state within a short period, ranging from minutes to hours.^6^ Activated microglia gain the ability to recruit neutrophil infiltration from the periphery and exacerbate neuronal damage by releasing pro-inflammatory factors, including interleukin 1 beta (IL-1β), tumor necrosis factor-alpha (TNF-α), and interleukin 6 (IL-6).^7^^,^^8^ It has been demonstrated that targeted depletion of resident microglia can attenuate cerebral ischemic damage during the early stages of stroke.^9^ Consequently, microglial activation assumes a crucial role in initiating the inflammatory cascade response and exacerbating brain injury following ischemic stroke.

Nuclear factor of activated T cells 5 (NFAT5), also known as tonicity responsive element enhancer binding protein (TonEBP) or osmotic response element binding protein (OREBP), is a novel member of the Rel transcription factor family.^10^ All members of the Rel transcription factor family possess a Rel homology domain that facilitates their interaction with the DNA of the target gene, thereby transcriptionally regulating the expression of the target gene.^11^^,^^12^ Cheung’s work has reported that knocking down NFAT5 leads to decreased macrophage infiltration in the adipose tissue of mice.^13^ Additionally, Shin et al have discovered that NFAT5^+/−^ mice exhibit a striking alleviation of neuroinflammatory responses and blood–brain barrier disruption in kainic acid-induced seizures.^14^ These findings suggest that NFAT5 may play a pro-inflammatory role in inflammatory response. Nevertheless, the precise mechanism underlying the pro-inflammatory function of NFAT5 remains to be elucidated. A previous study noted the up-regulation of NFAT5 expression in microglia in response to middle cerebral artery occlusion (MCAO) modeling.^15^ However, the role of microglial NFAT5 in neuroinflammation following MCAO modeling remains unclear.

NOD-like receptor pyrin domain containing 6 (NLRP6), a novel member of the NOD-like receptor (NLR) family, is classified as an intracytoplasmic pattern recognition receptor.^16^ Like other NLR members, NLRP6 has been reported to recruit apoptosis-associated speck-like proteins (ASC), which contain the CARD structural domain, in response to inflammatory stimuli.^17^ The CARD domain of ASC further interacts with the CARD structural domain of cysteine asparaginase (pro-caspase-1), leading to the activation of the NLRP6 inflammasome.^18^ The NLRP6 inflammasome, upon activation, initiates the cleavage of pro-caspase-1 into cleaved caspase-1, which subsequently leads to the cleavage of pro-IL-1β to mature IL-1β, thereby amplifying the inflammatory response.^19^ Our published work has demonstrated that activation of the NLRP6 inflammasome exacerbates neuroinflammation and aggravates inflammatory injury after MCAO modeling.^20^ However, whether and how NFAT5 regulates the NLRP6 inflammasome are still poorly understood. In this work, we aimed to investigate the role and mechanism of NFAT5 in microglia-mediated neuroinflammation following MCAO modeling.

## Materials and methods

### Mice

All C57BL/6 mice were obtained from Chongqing Medical University under the approval of the Institutional Animal Care and Use Committee. All mice involved in this experiment were cared for in strict accordance with the Guidelines for Laboratory Animal Care and Use Committee (NIH Publication No. 85-23, revised 1996). All mice were maintained at a constant temperature (25 °C–26 °C) with adequate food and water. Every effort was made to avoid pain and unintentional death.

### MCAO modeling

The middle cerebral artery occlusion (MCAO) modeling was performed as previously mentioned.^21^ Briefly, male mice (8–10 weeks, 20–25 g) were anesthetized with 3% isoflurane and maintained with 1.5% isoflurane. The left common carotid, internal carotid, and external carotid arteries were exposed and gently dissected. A threaded plug with a 0.20-mm diameter silicone head was then guided into the internal carotid artery via the common carotid artery and moved slowly to block the middle cerebral artery. After 1 h, the threaded plug was removed and the internal carotid artery was reperfused for 24 h. In the sham group, all procedures were identical except for the insertion of the sheath. A homeothermic heating pad was used to monitor core body temperature during anesthesia and maintain it at 37 °C.

### Cell exposure

BV2 (a commonly used mouse microglial line), HT22 (mouse hippocampal neuronal cells), HEK 293T (human embryonic kidney cells), and N2A (mouse neuroblastoma N2a cells) were obtained from the National Infrastructure of Cell Line Resource of China. These cells were maintained in Dulbecco’s modified Eagle’s medium/high glucose (DMEM; Gibco, Thermo Fisher Scientific, New York, USA) complemented with 10% fetal bovine serum (VivaCell, Shanghai, China), 100 U/mL penicillin, and 100 g/mL streptomycin (Thermo Fisher Scientific, USA) in 5% CO_2_ humidified air at 37 °C.

### OGD/R modeling

Oxygen and glucose deprivation/reoxygenation (OGD/R) modeling was performed as described previously. Briefly, BV2 cells were gently rinsed three times with phosphate-buffered saline and cultivated in glucose-free DMEM without fetal bovine serum in a hypoxia incubator (Gibco, Thermo Fisher Scientific, New York, USA) perfused with 1% O_2_ and 5% CO_2_.^22^ After 4 h, the culture medium was replaced with DMEM/high glucose containing 10% fetal bovine serum for 24 h.

### Collection of conditioned media

After 24 h of the OGD/R modeling, conditioned medium was obtained from BV2 with or without OGD/R treatment, centrifuged at 5000 *g* for 5 min, shortly stored at 4 °C, and administered into HT22 within 24 h. Specifically, the conditioned media were mixed with fresh DMEM and treated with HT22.^23^ The control group was maintained with fresh DMEM only.

### Quantitative PCR

Total RNA was isolated with Trizol buffer (Invitrogen, Thermo Fisher Scientific, USA). 800 ng of RNA were reverse-transcribed into cDNA using ABScript RT Master Mix for quantitative PCR with gDNA Remover (ABclonal, Wuhan, China). Quantitative PCR was conducted with 2X Universal SYBR Green Fast qPCR Mix (ABclonal, Wuhan, China). Relative levels were quantified using the 2^−ΔΔCT^ assay, normalized to β-actin. The primers used in this article are listed in Table 1.

**Table 1:** Sequences of primers for quantitative PCR.

Species	Gene	Sequence (5′—3′)
Mouse	*Nlrp6*	Forward primer:CGGGACGAGAGGAAGGCAGAG Reverse primer:CACACGATCCAGCACACGAAGG
Mouse	*Actin*	Forward primer:TGTCGAGTCGCGTCCACC Reverse primer:TCGTCATCCATGGCGAACTGG

### Magnetic resonance imaging

At 24 h after ischemia/reperfusion, mice were anesthetized with 1%–3% isoflurane and scanned by magnetic resonance imaging (7-T small animal MRI system, Bruker, Germany) while monitoring heart rate, respiratory rate, and body temperature (SurgiVet V3395TPR, Smiths Medical, USA). In addition, T2-weighted images were calculated by the 3D Slicer (5.22 version) to measure infarct volume. The total infarct volume of each brain was computed by totaling the infarcted volumes of all brain slices [infarcted area (mm^2^) × thickness (0.60 mm)].^24^

### Measurement of grip force

Using a grip strength meter, the mice’s whole-limb strength was measured 24 h after ischemia by gently lifting the tails and allowing the mice to grasp a rigid bar attached to a sensor and a digital force gauge. When the first active grasp was demonstrated, the mouse was pulled backward in a horizontal plane with increasing force until the grasp was overcome. The maximum and mean force that the mouse applied to the rigid bar before releasing the grip was recorded.^25^ In each mouse, three trials were performed, with a minimum of 10 min between each one.

### Immunofluorescence staining

Mice were euthanized and sacrificed 24 h after reperfusion. Brains were perfused with saline and fixed in 4% paraformaldehyde solution for 24 h, and then dehydrated in 20% and 30% sucrose for 3 days to complete immersion. The brains were frozen in a cryostat microtome and sliced into 8-μm thick sections in the coronal plane. After the sections were permeabilized and blocked with 5% goat serum (with 0.5% Triton X-100) for 1 h, the sections were rinsed with phosphate-buffered saline and then probed with primary antibody at 4 °C for 12 h. The sections were then rinsed with phosphate-buffered saline, followed by incubation with the appropriate secondary antibody at 37 °C for 1 h, stained with DAPI, and observed by immunofluorescence microphotography.

### Hematoxylin and eosin staining

Mice were euthanized and sacrificed at 24 h after reperfusion. After the brains were perfused with 0.9% saline from the heart, the brains were infused with 4% paraformaldehyde for internal fixation, followed by immersion in 4% paraformaldehyde for 48 h for external fixation. The brains were then dehydrated through a series of alcohol gradients, cleared in dimethylbenzene, embedded in wax, and sliced into 5 μm-thick sections. Before immunostaining, brain sections were dewaxed in dimethylbenzene, debenzolized through decreasing concentrations of alcohol, and rinsed with phosphate-buffered saline. After staining with hematoxylin and eosin, the brain sections were dehydrated, cleared, and observed under a bright-field microscope.

### Nissel staining

Brain sections were prepared as described above. After debenzolization and dewaxing, brain sections were stained in a tar violet solution (Sigma–Aldrich, USA) for 15 min. The brain sections were then subjected to a color separation reaction and dehydrated in graded ethanol. Finally, the brain sections were coverslipped, sealed, and examined under a microscope.

### TUNEL staining

The TUNEL (TdT-mediated dUTP nick-end labeling) Kit (Servicebio Technology, Wuhan, China) was used to detect the apoptosis of brain tissues. For this experiment, dewaxed and debenzolized brain sections were stained with TUNEL reaction mixture in the dark at 37 °C for 60 min, and visualized by fluorescence microscopy.

### Chromatin immunoprecipitation

The chromatin immunoprecipitation (ChIP) was performed using the SimpleChIP ® Enzymatic Chromatin IP Kit (CST#9002, Boston, USA). Briefly, after fixing with 1% formaldehyde for 5 min and washing with phosphate-buffered saline, approximately 2 × 10^7^ BV2 cells were collected in a 10 cm^2^ cell culture dish. The cell pellet was lysed with cell lysis buffer, followed by digestion with micrococcal nuclease. After the cell lysate was sonicated to obtain chromatin fragments, the protein lysate was immunoprecipitated with NFAT5 antibody overnight at 4 °C. The next day, antibody-chromatin precipitates were combined with agarose beads at 4 °C for 2 h. The ChIP Elution Buffer was mixed and heated to 65 °C for 0.5 h to separate DNA fragments from the beads-antibody-chromatin cross-link. After purification by a spin column, the DNA fragments were subjected to PCR and real-time quantitative PCR.

### Plasmid construction and transfection

We obtained full-length *Nfat5* sequences from the cDNA of BV2 cells and cloned them into Plenti6/TR vectors containing a flag tag with XbaI and XhoI restriction sites. After prediction of the potential binding site of NFAT5 to the *Nlrp6* promoter region using Jaspar 2020 (https://jaspar.genereg.net/), *Nlrp6* promoter, promoter segments and mutants obtained from BV2 genomic DNA templates were cloned into the pGL4.10 vector using KpnI and NheI restriction sites. The *Nlrp6* 3′UTR region and *Nlrp6* 5′UTR region were synthesized by BGI (Beijing Genomics Institute, Beijing, China). Plasmids were transiently transfected into HEK293T cells with polyethyleneimine linear (PEI) (polyscience, USA). The primers used for plasmid construction and sh-RNA sequences are shown in Table 2.

**Table 2:** Sequences of primers for plasmid construction.

Oligonucleotides	Forward primer (5′—3′)	Reverse primer (5′—3′)
*Nfat5*	GCTCTAGAATGCCCTCGGACTTCATCTCA	CCGCTCGAGAAAGGAGCCGGTTAAATTGTTCC
*Nlrp6* promoter	GGGGTACCTGGTCATATTTGTATGTCTGTGCC	CTAGCTAGCTAGCTTCCCTACGTGGGTCT
Fragment 1	GGGGTACCGCTTGGCTTTGTATTTCTTC	CTAGCTAGCTAGCTTCCCTACGTGGGTCT
Fragment 2	GGGGTACCGCCCTCTTGATTTTCACGCC	CTAGCTAGCTAGCTTCCCTACGTGGGTCT
Mutant primer 1	GGGGTACCTGGTCATATTTGTA	TTTCCCACAGGGGGAAGGCGAAGCCGTGTTTT
Mutant primer 2	ACACGGCTTCGCCTTCCCCCTGTGGGAAAGCCA	CTAGCTAGCTAGCTTCCCTACGT
Mutant primer 3	GGGGTACCTGGTCATATTTGTA	CTAGCTAGCTAGCTTCCCTACGT

### Lentiviral packaging and transfection

The scrambled sh-RNA, NFAT5 sh-RNA1, and NFAT5 sh-RNA2 lentiviruses were produced by pSIH1 lentiviral vectors with BamHI and EcoRI restriction sites and transfected into BV2 cells. Next, we obtained BV2 cells with stable NFAT5 knockdown by 2 μg/mL puromycin (MCE, USA) treatment. The sh-RNA sequences are listed in Table 3.

**Table 3:** Sequences of sh-RNA in BV2 cells.

Species	Name	Sequence (5′—3′)
Mouse	Scrambled shRNA	Sense: TTC TCC GAA CGT GTC ACG TTT Anti-sense: ACG TGA CAC GTT CGG AGA ATT
Mouse	Nfat5 sh-RNA 1	Sense: CCA GTT CCT ACA ATG ATA ACA CT Anti-sense: AGT GTT ATC ATT GTA GGA ACT GG
Mouse	Nfat5 sh-RNA 2	Sense: TGC GGA CAG TAT CCG GTT AAA Anti-sense: TTT AAC CGG ATA CTG TCC GCA

### Dual-luciferase reporter assay

The mouse NFAT5 plasmid (pLenti6-*Nfat5*-Flag) or an empty vector plasmid (TR), together with the pGL4.10 Nlrp6 promoter and the reference plasmid pGL4.74 (rLuc), were co-transfected into HEK293T and N2A for 48 h. Next, the cells were collected and their relative luciferase activity was assessed with a dual-luciferase reporter kit (HB-DLR-100, Hanheng, Shanghai, China) and detected using the GloMax 20/20 Luminometer (Promega, USA). Each assay was run at least three times.

### Adeno-associated virus (AAV) delivery

*Nfat5* shRNA and scrambled shRNA vectors driven under the F4/80 promoter followed by enhanced green fluorescent protein (EGFP) were constructed and packaged into an AAV9 by Genechem (Shanghai, China). The 3.5 μL AAV9-*Nfat5* shRNA (2.11 × 10^12^ vg/mL) and the 3.5 μL AAV9-scrambled shRNA (2 × 10^12^ vg/mL) were injected into the left lateral ventricle of mice (anteroposterior, −0.3 mm; mediolateral, +1.0 mm; dorsoventral, −2.5 mm) and 1 μL for the left hippocampus (anteroposterior, −2.0 mm; mediolateral, +1.5 mm; dorsoventral, −1.2 mm) at a rate of 0.2 μL/min 26. The MCAO model was generated by transfecting mice with AAV9-*Nfat5* shRNA and AAV9-scrambled shRNA for 28 days. The silencing efficiency of AAV9-*Nfat5* shRNA was determined by immunofluorescence. The shRNA sequences targeting mouse *Nfat5* and the scrambled shRNA sequence are shown in Table 4.

**Table 4:** The sequences of shRNA.

Species	Name	Sequence (5′—3′)
Mouse	*Nfat5* shRNA	Sense: CCA GTT CCT ACA ATG ATA ACA CT Anti-sense: AGT GTT ATC ATT GTA GGA ACT GG
Mouse	Scrambled shRNA	Sense: TTC TCC GAA CGT GTC ACG TTT Anti-sense: ACG TGA CAC GTT CGG AGA ATT

### Western blotting

Proteins from brain tissue and BV2 cells were analyzed by western blotting. After primary antibody probes and secondary antibody incubation, protein bands were visualized using enhanced chemiluminescence blotting detection reagents (Bio-Rad, USA). The antibodies incubated for western blotting are listed in Table 5.

**Table 5:** List of the antibodies for western blotting or immunofluorescence.

Antibody	Manufacturer	Catalogue number	Application	Dilution
NFAT5	Abcam, USA	ab3446	Immunofluorescence/chromatinimmunoprecipitation	1:100
NFAT5	Abcept	AP74036	Western blotting	1:500
NLRP6	Abclonal, China	A15628	Western blotting	1:1000
MPO	Abcam, USA	Ab208670	Immunofluorescence	1:200
Iba-1	Aifang, China	AF301643	Immunofluorescence	1:200
GFAP	Elabscience, China	E-AB70205	Immunofluorescence	1:200
NeuN	Abcam, USA	ab104224	Immunofluorescence	1:200
Bcl-2	Abclonal, China	A11313	Immunofluorescence	1:200
Bcl-2	Abclonal, China	A11313	Western blotting	1:500
Bax	Proteintech, USA	BS4084	Immunofluorescence	1:200
Bax	Proteintech, USA	BS4084	Western blotting	1:2000
ASC	CST, USA	D2W8U	Western blotting	1:1000
Pro-caspase-1	Abclonal, China	A0964	Western blotting	1:1000
Clevead-caspase-1	Affinity, USA	ab18256	Western blotting	1:500
Pro-IL-1β	Abclonal, China	A1112	Western blotting	1:1000
IL-1β	Affinity	AF4006	Western blotting	1:1000
IL-6	Santa, USA	SC-57315	Western blotting	1:200
TNF-α	Abclonal, China	A0277	Western blotting	1:1000
β-actin	Abclonal, China	AC026	Western blotting	1:20,000
Goat anti-rabbit secondary antibody	Thermo, USA	31,460	Western blotting	1:10,000
Goat anti-mouse secondary antibody	Thermo, USA	31,430	Western blotting	1:10,000

### CCK8

The viability of HT22 cells was analyzed by cell counting kit-8 (C0005, TargetMol, USA). In this experiment, HT22 cells were plated at a density of 3 × 10^3^ cells/well in 100 μL medium in 96-well microplates (BioFil, Guangzhou, China). The next day, the conditioned medium was administrated into HT22 cells. After 24 h of treatment, the conditioned medium was removed and replaced with a fresh medium. Then, 10 μL CCK-8 reagent was supplemented and cultivated for 2 h. Absorbance was recorded with a microtiter plate reader (Bio-Rad, USA) at 450 nm. Cell viability was calculated as per the following equation: cell viability = optical density (treatments-blank)/optical density (controls-blank) × 100%.

### LDH analysis

After treatment with a conditioned medium for 24 h, the culture medium of HT22 cells was collected to evaluate the release of lactate dehydrogenase (LDH) in the culture medium (Beyotime, China). The absorbance data were measured using a 96-well plate reader (BioFil, China) at 450 nm.

### Annexin V-FITC and propidium iodide dual staining assay

Apoptosis of HT22 cells treated with different BV2 cell-conditioned medium was performed using an annexin V-FITC/propidium iodide dual staining assay (Beyotime Biotechnology, Shanghai, China). After HT22 cells were treated with conditioned medium for 24 h, 1 × 10^6^ cells from each group were collected and stained with annexin V-FITC and propidium iodide at 4 °C for 30 min. The fluorescence intensity of the stained cells was then analyzed by flow cytometry (FACS Vantage SE, Becton Dickinson, San Jose, CA, USA), and FlowJo (BD Biosciences, Wokingham, UK) was used for data processing.

### ELISA

BV2 medium was collected at 24 h with or without OGD/R treatment, centrifuged at 5000 *g* and 4 °C for 5 min, and stored at −80 °C. The concentrations of IL-1β, IL-6, and TNF-α in the BV2 medium were examined with enzyme-linked immunosorbent assay (ELISA) Kits (Jiangsu Meibiao Biotechnology Co., Ltd., Jiangsu, China).

### Administration of actinomycin D

Actinomycin D (Aladdin, Shanghai, China) was used to block the mRNA synthesis of BV2 cells. After 24 h of OGD/R, 5 μg/mL actinomycin D was administered into BV2 cells for 0 h, 1 h, 2 h, 4 h, and 6 h. These BV2 cells were then harvested for quantitative PCR to determine the half-life of *Nlrp6* mRNA.

### Statistical analysis

GraphPad Prism software (version 8.0) was used for statistical analysis. Initially, we used the Shapiro–Wilk test to assess the normal distribution and Bartlett’s test to assess the homogeneity of variance. If the data exhibited a normal distribution and homogeneity of variance, the student’s *t*-test was used to analyze data in two groups, while one-way ANOVA and Tukey’s multiple comparison test was used to analyze data between more than two groups. Otherwise, analysis of the non-parametric data was performed using the Mann–Whitney test in two groups.

## Results

### Increased expression of NFAT5 in microglia after OGD/R and MCAO modeling

**Figure 1:**
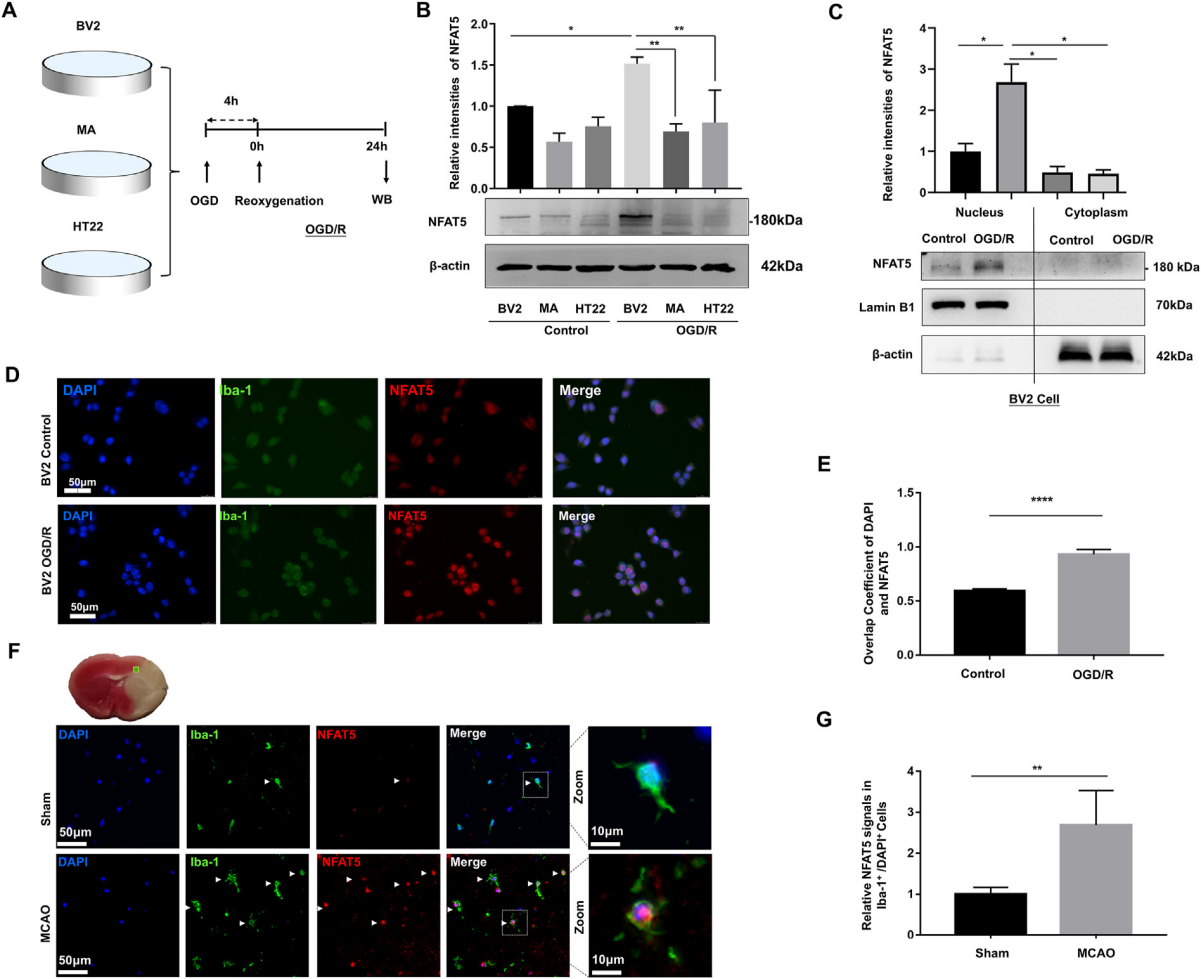
Increased expression of NFAT5 in microglia after OGD/R and MCAO modeling. **(A)** Schematic of three cell lines undergoing OGD/R modeling. BV2, mouse microglia cell line; MA, mouse astrocyte cell line; HT22, mouse hippocampal neuron cell line. **(B)** Western blots for NFAT5 in BV2, MA, and HT22 cell lines after OGD/R modeling (*n* = 3). **(C)** The NFAT5 protein level in the nuclei and cytoplasm of BV2 cells was detected by western blotting (*n* = 3). **(D)** Representative immunofluorescence images of NFAT5 in BV2 cells (bar = 25 μm). **(E)** The overlap coefficient of DAPI and NFAT5 in BV2 cells (*n* = 3). **(F, G)** Immunofluorescence for NFAT5 in microglia from peri-infarct brain tissues of mice (bar = 50 μm). Iba-1 was marked in green for microglia, and NFAT5 was marked in red. The cells indicated by the white arrows were microglia. Scale bar in magnified view: 10 μm. The data were presented as mean with standard deviation. ∗*p* < 0.05, ∗∗*p* < 0.01, and ∗∗∗∗*p* < 0.0001.

It has been widely reported that microglia are activated within 24 h in the acute stage, causing a strong inflammatory response.^27^ Therefore, we collected the samples for subsequent analysis 24 h following OGD/R and MCAO modeling. To examine the expression of NFAT5 in three types of cells (microglia BV2 cell line, astrocytes MA cell line, and hippocampal neurons HT22 cell line), we subjected BV2, MA, and HT22 to the OGD/R model and detected the expression level of NFAT5 by western blotting assay. As illustrated in Figure 1A and B, NFAT5 was expressed in MA, HT22, and BV2 cell lines. Notably, the expression of NFAT5 in BV2 cells was elevated after OGD/R modeling. In addition, the protein level of NFAT5 in BV2 was higher than that in HT22 and MA after OGD/R modeling. Then, we isolated the nucleus and cytoplasm of BV2 cells and assessed the protein level of NFAT5 using western blotting. Our results indicated that NFAT5 was predominantly localized in the nucleus and increased after OGD/R modeling (Fig. 1C). The immunofluorescence staining results revealed that NFAT5 was primarily colocalized with the nucleus, and the coefficient of NFAT5 and DAPI colocalization increased after OGD/R modeling (Fig. 1D, E). Moreover, we labeled microglia with immunofluorescence to investigate the expression of microglial NFAT5 in mouse brain tissue after MCAO modeling. The results indicated that NFAT5 was localized in the nucleus of microglia. Compared with the sham group, the fluorescence signal of microglial NFAT5 in the MCAO group was significantly enhanced (Fig. 1F, G). In summary, these results suggested that the expression of NFAT5 in microglia was elevated after OGD/R modeling and MCAO modeling.

### Rescue of MCAO-induced cerebral infarction and neurological deficits in mice through microglial NFAT5 interference

**Figure 2:**
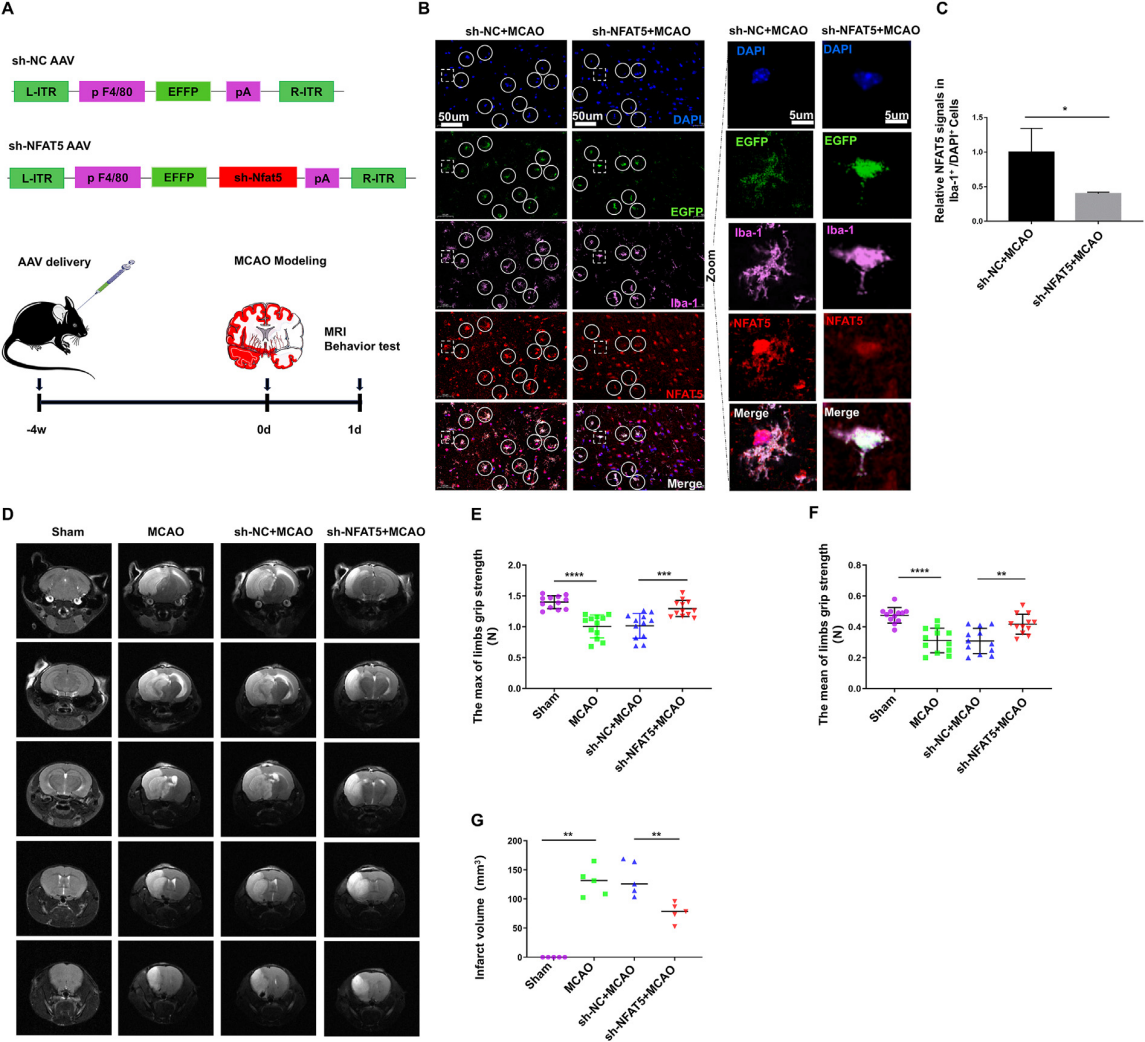
Rescue of MCAO-induced cerebral infarction and neurological deficits in mice through microglial NFAT5 interference. **(A)** Design of adeno-associated virus (AAV) to knock down microglial NFAT5. **(B, C)** Representative immunofluorescence images depicting Iba-1 (in pink) and NFAT5 (in red) (B) and quantification of NFAT5 fluorescence intensity in Iba-1 positive cells (C). The AAV expressed enhanced green fluorescent protein (EGFP). The infected microglia were delineated with circular and square annotations. The magnified view focused on the microglia within the square annotation. Scale bar: 50 μm. Scale bar in magnified view: 5 μm. The data were presented as mean with standard deviation (*n* = 3). **(D, G)** Representative images and quantification of magnetic resonance imaging data. The data were presented as median (*n* = 5). **(E, F)** Maximum and mean mouse limb grip strength (*n* = 12). The data were presented as mean with standard deviation. ∗*p* < 0.05, ∗∗*p* < 0.01, ∗∗∗*p* < 0.001, and ∗∗∗∗*p* < 0.0001.

In this study, we employed a recombinant adeno-associated virus (rAAV) carrying a microglia-specific promoter (F4/80) to selectively suppress the expression of NFAT5 in microglial cells (Fig. 2A). To validate the specificity of rAAV for microglia, we conducted immunofluorescence experiments to examine the cellular localization of EGFP encoded by rAAV. As illustrated in Figure S1, we observed co-localization of EGFP with Iba-1-positive cells, whereas NeuN-positive and GFAP-positive cells did not exhibit co-localization with EGFP. This observation confirms the precise targeting of sh-NFAT5 rAAV to microglia. Subsequently, we quantified the fluorescent signal intensity of NFAT5 within Iba-1-positive cells to assess the knockdown efficacy of sh-NFAT5 rAAV in microglia. Our results demonstrate a significant reduction in NFAT5 signal intensity in the sh-NFAT5 group compared with the sh-NC group (Fig. 2B, C), confirming the successful knockdown of NFAT5.

To explore the role of microglial NFAT5 in ischemic stroke, we evaluated brain infarct volumes in mice using magnetic resonance imaging. The T2W phase indicated that normal brain tissue exhibited low signal intensity, while infarcted brain tissue displayed high signal intensity. As shown in Figure 2D and G, the MCAO modeling induced substantial brain infarction, whereas specific NFAT5 knockdown significantly reversed MCAO-induced cerebral infarction. This suggests that microglial NFAT5 knockdown effectively mitigates the extent of brain infarction following MCAO modeling. Patients with cerebral infarction often exhibit symptoms of impaired limb mobility. Thus, we measured the grip strength of mice after MCAO model induction. Our data indicate a significant impairment in both maximum and mean grip force after MCAO modeling. Notably, sh-NFAT5 greatly enhanced both the maximum and mean grip force values (Fig. 2E, F). In summary, our findings demonstrate that the inhibition of microglial NFAT5 can alleviate brain tissue morphological damage and ameliorate the deterioration of neurological deficits caused by MCAO modeling in mice.

### Microglial NFAT5 knockdown mitigates MCAO-induced brain morphological damage and apoptosis

**Figure 3:**
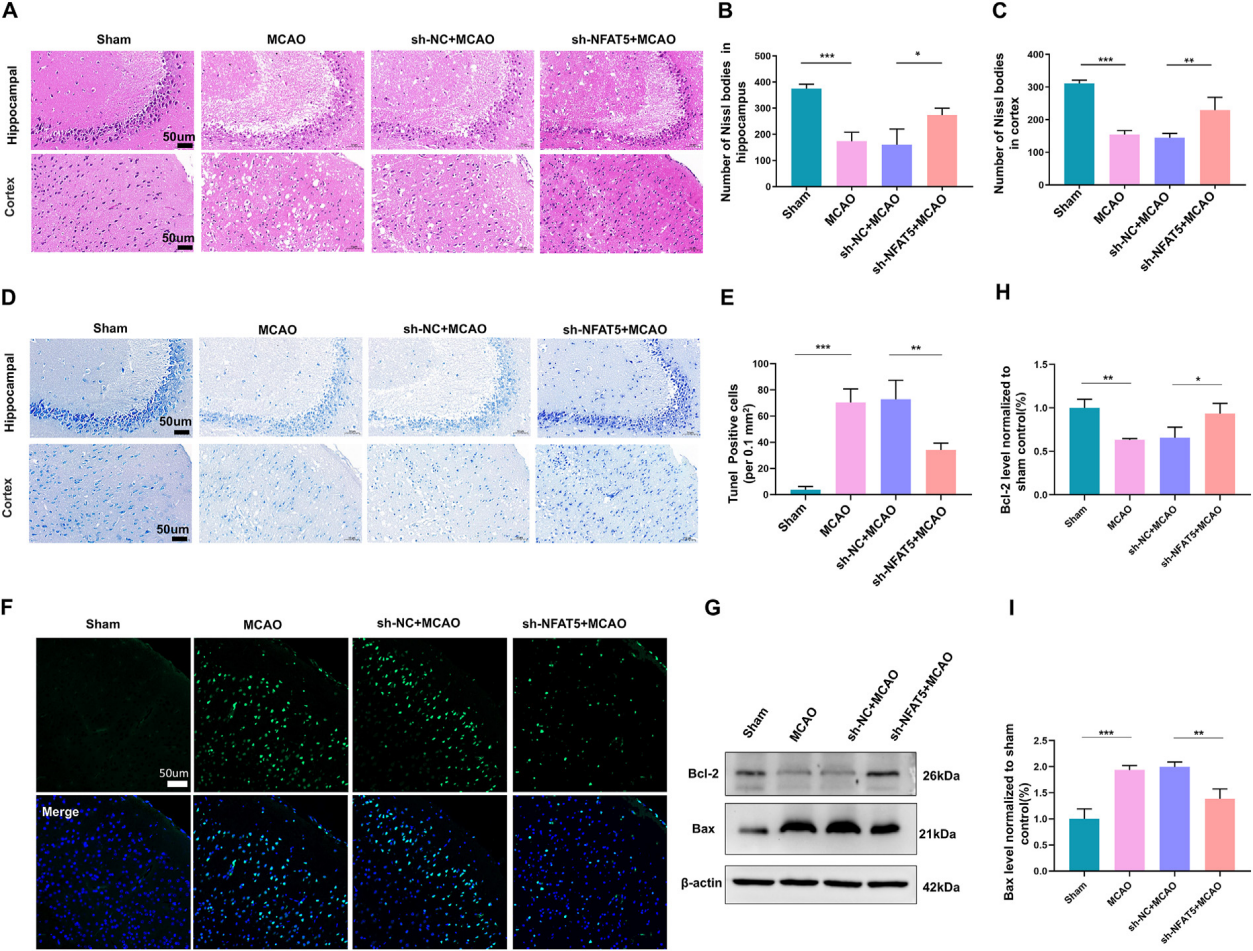
Microglial NFAT5 knockdown mitigates MCAO-induced brain morphological damage and apoptosis. **(A)** Representative images of hematoxylin-eosin staining in the hippocampus and cortex of mice. Scale bar: 50 μm (*n* = 3). **(B**–**D)** Representative images of Nissel staining (D) and quantification of Nissel-positive cells in the hippocampus (B) and cortex (C). Scale bar: 50 μm. The data were presented as mean with standard deviation (*n* = 3). **(E, F)** Representative TUNEL assay images in cortical brain tissue regions (F) and quantification of TUNEL-positive cells per 0.1 mm^2^ (E). TUNEL staining is shown in green, and nuclei are labeled in blue (*n* = 3). Scale bar: 50 μm. **(G**–**I)** Western blotting analysis of Bcl-2 and Bax expression levels (*n* = 3). Data presented as means with standard deviation. ∗*p* < 0.05, ∗∗*p* < 0.01, and ∗∗∗*p* < 0.001.

Next, we examined the morphological damage in the brain tissue using hematoxylin-eosin staining and Nissl staining. Consistently, we observed disorganization and a sparse structure in the hippocampus and cerebral cortex (Fig. 3A, D). Numerous Nissl bodies were observed in the hippocampal and cortex of sham-operated mice. There was a reduction in the number of Nissl bodies that appeared to disintegrate or disappear in the MCAO mice. However, these alterations were markedly ameliorated by microglial NFAT5 knockdown. Additionally, we quantified Nissl bodies in the hippocampus and cerebral cortex, revealing a substantial decrease in their numbers following MCAO modeling, which was subsequently improved by silencing microglial NFAT5 (Fig. 3B, C).

To assess the impact of microglial NFAT5 on apoptosis following MCAO modeling, we conducted TUNEL staining and quantification to evaluate apoptosis around the penumbra region. The penumbra region in brain tissue from MCAO mice exhibited a significant increase in apoptosis, while microglial NFAT5 knockdown reduced MCAO-induced apoptosis (Fig. 3E, F). Furthermore, we performed western blotting analysis to assess the expression levels of apoptosis-associated proteins, specifically BCL2-associated X protein (Bax) and B cell leukemia/lymphoma 2 (Bcl-2), in brain tissue. Our observations revealed that the Bcl-2 level significantly decreased, while the level of Bax considerably increased following MCAO modeling. However, the inhibition of microglial NFAT5 markedly increased the expression of Bcl-2 and prominently reduced the expression of Bax under MCAO conditions (Fig. 3G–I). These results collectively suggest that microglial NFAT5 may play a crucial role in modulating apoptosis in brain tissue following MCAO modeling.

### Microglial NFAT5 silencing attenuates neuronal apoptosis in OGD/R model

**Figure 4:**
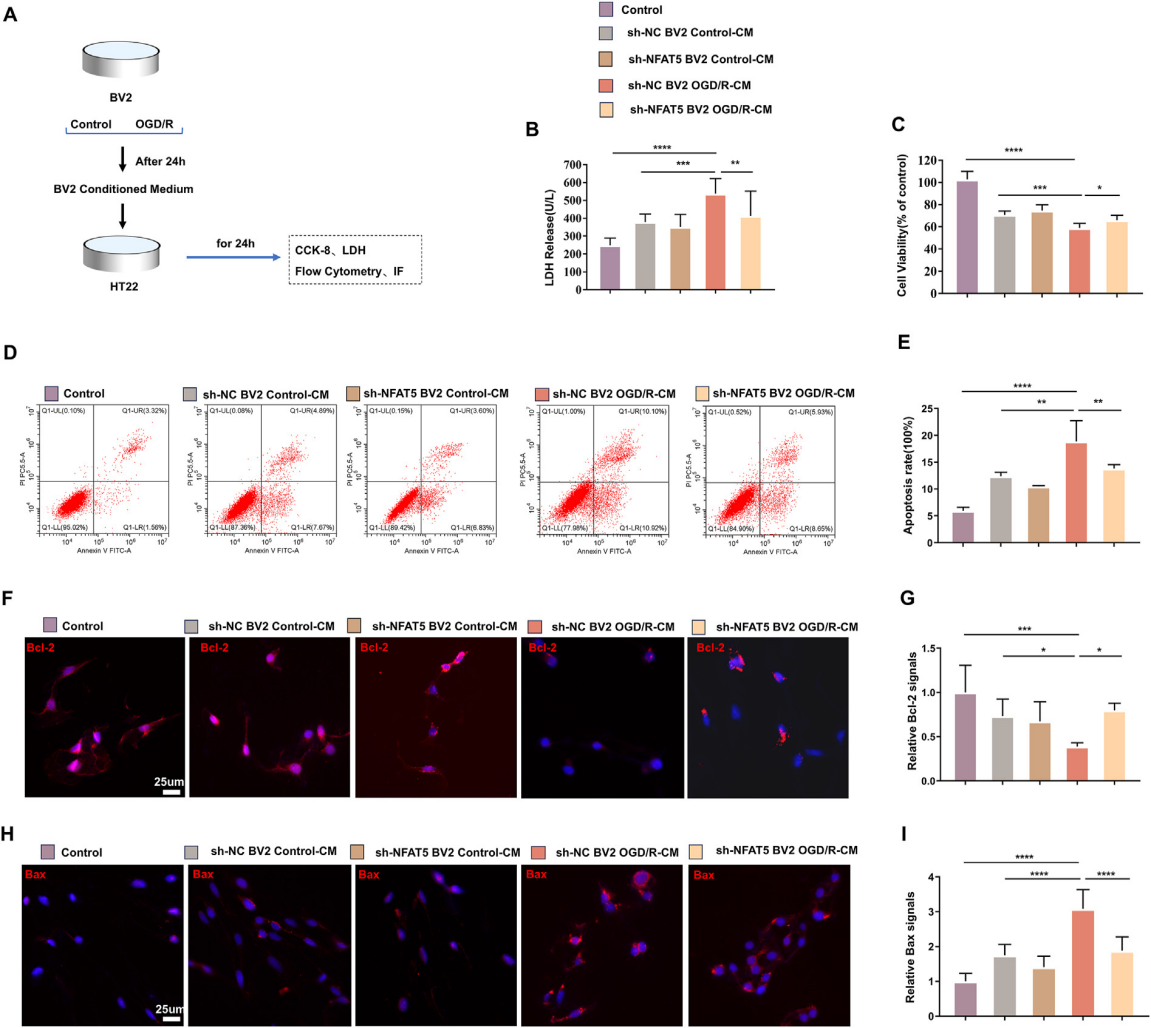
Microglial NFAT5 silencing attenuates neuronal apoptosis in OGD/R model. **(A)** Schematic representation of BV2 (mouse microglia cell line) conditioned medium treatment on HT22 (mouse hippocampal neuron cell line). **(B)** A lactate dehydrogenase (LDH) assay was used to detect the released LDH in HT22 medium (*n* = 8–12). (**C**) CCK-8 assay was used to measure the cell survival rate of HT22 (*n* = 9). **(D, E)** Representative images and quantification of flow cytometry illustrating the percentage of annexin V-FITC and propidium iodide (PI)-labeled HT22 cells (*n* = 3). **(F–I)** Representative immunofluorescence images of Bcl-2 (F) and Bax (H) and quantification of fluorescence intensity for Bcl-2 (G) and Bax (I) in HT22 cells (*n* = 3). Scale bar: 25 μm. The data were presented as mean with standard deviation. ∗*p* < 0.05, ∗∗*p* < 0.01, ∗∗∗*p* < 0.001, and ∗∗∗∗*p* < 0.0001.

We further determined the impact of microglial NFAT5 on neuronal apoptosis *in vitro*. Initially, we established two stable BV2 (mouse microglia cell line) cell lines with NFAT5 knockdown using sh-RNA. Western blotting analysis confirmed a consistent decrease in NFAT5 levels in both sh-RNA1 (sh-1) and sh-RNA2 (sh-2) cell lines, with sh-2 exhibiting distinct cellular characteristics such as rounder cells and shorter cell protrusions compared with wild-type and sh-NC BV2 cells (Fig. S2A, B). Consequently, we selected the sh-1 cell line for subsequent experiments. We then created an *in vitro* model using a microglia-conditioned medium subjected to OGD/R to simulate the interaction between microglia and neurons (Fig. 4A). To assess neuronal apoptosis in HT22 (mouse hippocampal neuronal cells), we conducted flow cytometry using annexin V-FITC and propidium iodide staining, as well as immunofluorescence staining for Bcl-2 and Bax. Our results demonstrated that the BV2 OGD/R-conditioned medium exacerbated HT22 apoptosis, while genetic inhibition of NFAT5 suppressed OGD/R-induced HT22 apoptosis (Fig. 4D–I). Furthermore, CCK-8 assay results revealed a significant decrease in HT22 cell survival when exposed to OGD/R-conditioned medium, whereas microglial NFAT5 knockdown increased HT22 cell survival (Fig. 4C). Additionally, LDH assay results indicated that OGD/R-conditioned medium significantly elevated LDH levels, while BV2 NFAT5 knockdown mitigated LDH content in HT22 cells (Fig. 4B). Collectively, these findings suggest that microglial NFAT5 may contribute to neuronal apoptosis and injury following BV2 OGD/R-conditioned medium exposure.

### NFAT5 inhibition ameliorates microglia-mediated neuroinflammation in MCAO and OGD/R models

**Figure 5:**
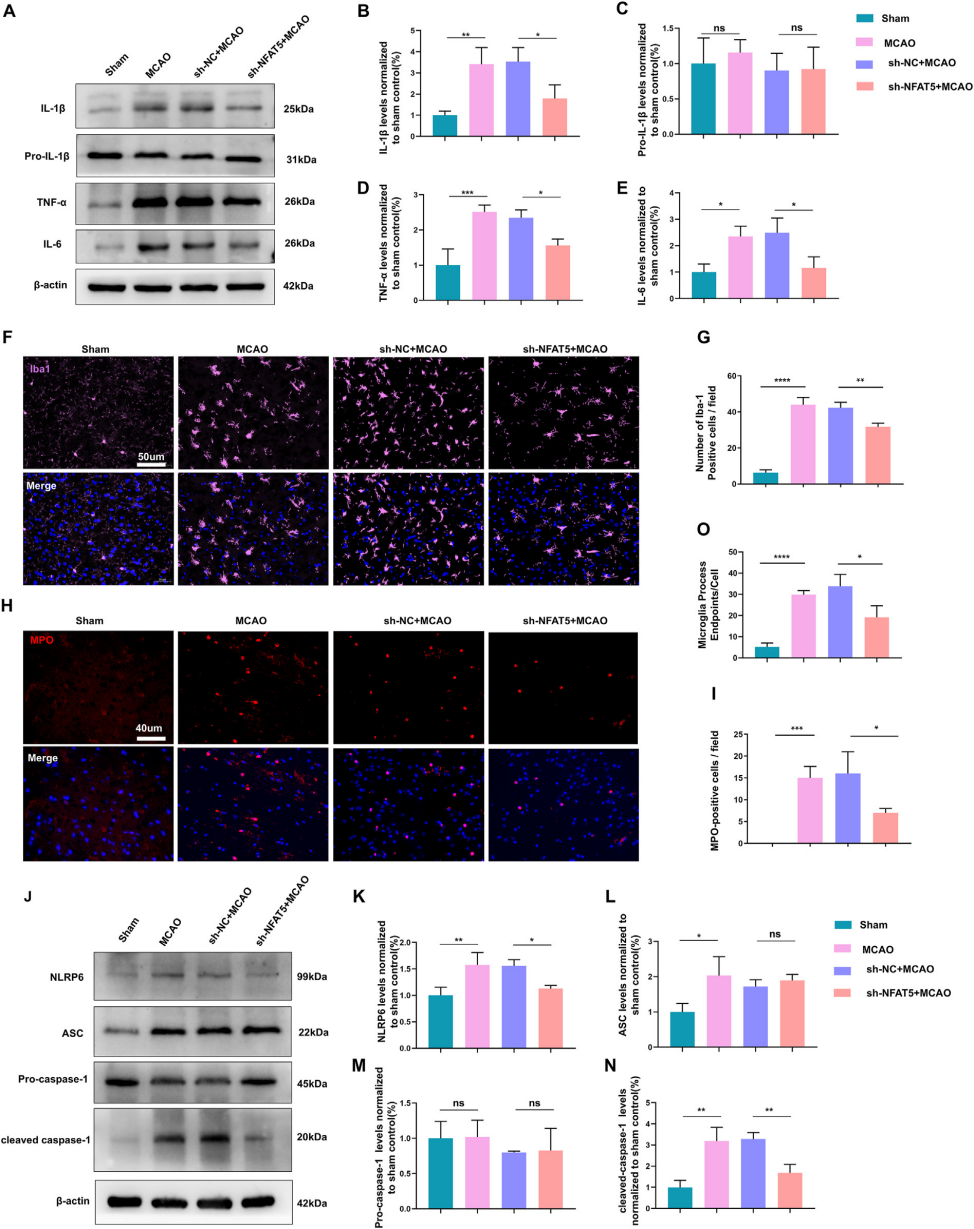
NFAT5 inhibition ameliorates microglia-mediated neuroinflammation and NLRP6 inflammasome activation in MCAO model. **(A**–**E)** Western blotting analysis of pro-inflammatory factor protein levels (IL-1β, TNF-α, and IL-6) in brain tissues (A), with protein levels normalized to the sham group (B–E) (*n* = 3). **(F, G)** Representative immunofluorescence images of Iba-1 (F) and quantification of Iba-1-positive cells (G) in brain sections (*n* = 3). Scale bar: 50 μm. **(H, I)** Representative immunofluorescence images of MPO (H) and quantification of MPO-positive cells (I) in brain sections (*n* = 3). Scale bar: 40 μm. **(J**–**N)** Western blotting analysis was applied for NLRP6, ASC, pro-caspase-1, and cleaved-caspase-1 in brain tissue and the protein levels were normalized to the sham group (*n* = 3). **(O)** Quantification of microglial morphology (*n* = 3). The data were presented as mean with standard deviation. ∗*p* < 0.05, ∗∗*p* < 0.01, ∗∗∗*p* < 0.001, and ∗∗∗∗*p* < 0.0001; ns, no statistical significance.

Considering the pivotal role of microglia in neuroinflammation following ischemic stroke through the production of pro-inflammatory factors such as IL-1β, TNF-α, and IL-6,^28^ we assessed the expression of these pro-inflammatory factors in both MCAO and OGD/R models. Our western blotting results demonstrated that the MCAO model led to increased expression of IL-1β, TNF-α, and IL-6, while the suppression of NFAT5 significantly reduced these pro-inflammatory factor levels (Fig. 5A, B, D, E). Furthermore, we employed immunofluorescence to investigate microglial activation and neutrophil infiltration. Activated microglia were identified by Iba-1 labeling (pink), while neutrophils were labeled with MPO (red). Additionally, we quantified microglial morphology by Image J (https://imagej.net/AnalyzeSkeleton). Microglia and process endpoints increased after MCAO modeling. However, microglial NFAT5 knockdown reduced microglia and process endpoints compared with the sh-NC + MCAO group (Fig. 5F, G, O). Our immunofluorescence results provided compelling evidence that microglial activation and neutrophil infiltration were amplified following MCAO modeling around the penumbra region, but microglial NFAT5 knockdown attenuated both microglial activation and neutrophil infiltration (Fig. 5F–I).

**Figure 6:**
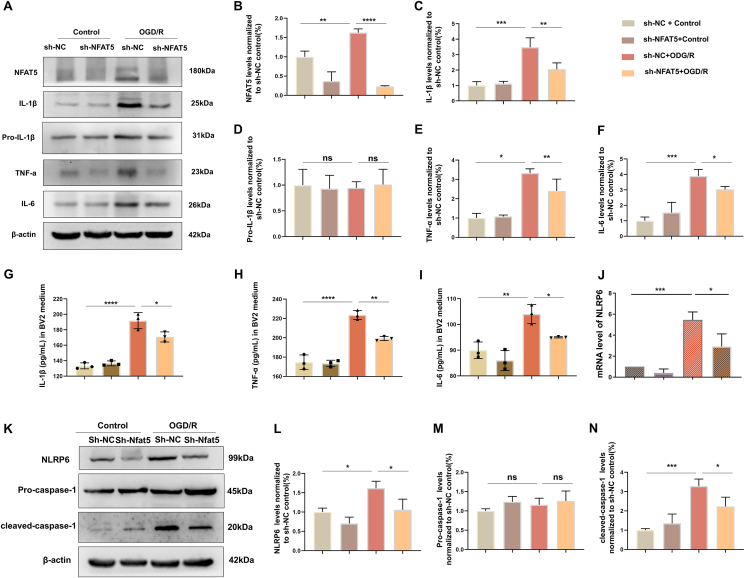
NFAT5 silencing suppresses inflammatory response and NLRP6 inflammasome activation in OGD/R Model. **(A**–**F)** Western blotting analysis of NFAT5, pro-IL-1β, IL-1β, TNF-α, and IL-6 protein levels in BV2 cells (A), with the protein levels normalized to the sh-NC control group (B–F) (*n* = 3). **(G**–**I)** ELISA measurement of IL-1β, TNF-α, and IL-6 concentrations in BV2 cell culture medium (*n* = 3). **(K–N)** Western blotting analysis was performed for NLRP6, pro-caspase-1, and cleaved-caspase-1 in BV2 cells, with the protein levels normalized to the sh-NC control group (*n* = 3). **(J)** The *Nlrp6* mRNA level in BV2 cells was detected by quantitative PCR (*n* = 3). The data were presented as mean with standard deviation. ∗*p* < 0.05, ∗∗*p* < 0.01, ∗∗∗*p* < 0.001, and ∗∗∗∗*p* < 0.0001; ns, no statistical significance.

To further evaluate the levels of TNF-α, IL-1β, and IL-6 *in vitro*, we collected BV2 cells and their culture medium for western blotting and ELISA analysis. In comparison to the sh-NC control group, OGD/R significantly elevated the protein levels of pro-inflammatory factors, while silencing NFAT5 inhibited their expression (Fig. 6A–F). Additionally, ELISA results were consistent with the western blotting findings, indicating that OGD/R modeling increased the secretion of pro-inflammatory factors. Conversely, NFAT5 knockdown reduced OGD/R-induced secretion of TNF-α, IL-1β, and IL-6 (Fig. 6G–I). Notably, NFAT5 did not significantly alter the levels of pro-IL-1β (Figure 5, Figure 6D). In summary, our *in vitro* and *in vivo* findings collectively suggest that NFAT5 may exacerbate microglia-mediated neuroinflammation in both MCAO and OGD/R models.

### NFAT5 silencing suppresses NLRP6 inflammasome activation *in vivo* and *in vitro*

NLRP6, a recent focus in the NLR family, has garnered significant attention in recent years.^29^ Our previously published research has confirmed that NLRP6 inflammasome activation contributes to inflammatory injury following ischemic stroke.^20^ Given the established role of NFAT5 in neuroinflammation, we sought to investigate whether NFAT5 mediated the NLRP6 inflammasome. Thus, we assessed the protein levels of NLRP6, ASC, pro-caspase-1, and cleaved-caspase-1 through western blotting analysis. Remarkably, we observed elevated levels of NLRP6, ASC, and cleaved-caspase-1 following MCAO modeling. Conversely, the knockdown of microglial NFAT5 suppressed NLRP6 levels and caspase-1 activation in the *in vivo* MCAO model (Fig. 5J–N). However, the protein levels of pro-caspase-1 and ASC remained largely unaffected. Furthermore, we evaluated the protein level of NLRP6 and caspase-1 activation in BV2 cells. Consistent with our *in vivo* results, the *in vitro* findings indicated that OGD/R modeling led to an up-regulation of NLRP6 levels and caspase-1 activation, whereas NFAT5 silencing inhibited NLRP6 levels and caspase-1 activation (Fig. 6K–N). Subsequently, quantitative PCR results revealed a significant increase in NLRP6 mRNA levels following OGD/R modeling, which was markedly decreased upon NFAT5 knockdown (Fig. 6J). In summary, these findings collectively demonstrate that NFAT5 regulates both the mRNA and protein levels of NLRP6 and NLRP6 inflammasome activation in both MCAO and OGD/R models.

### NFAT5 is a transcription factor for the Nlrp6 promoter

**Figure 7:**
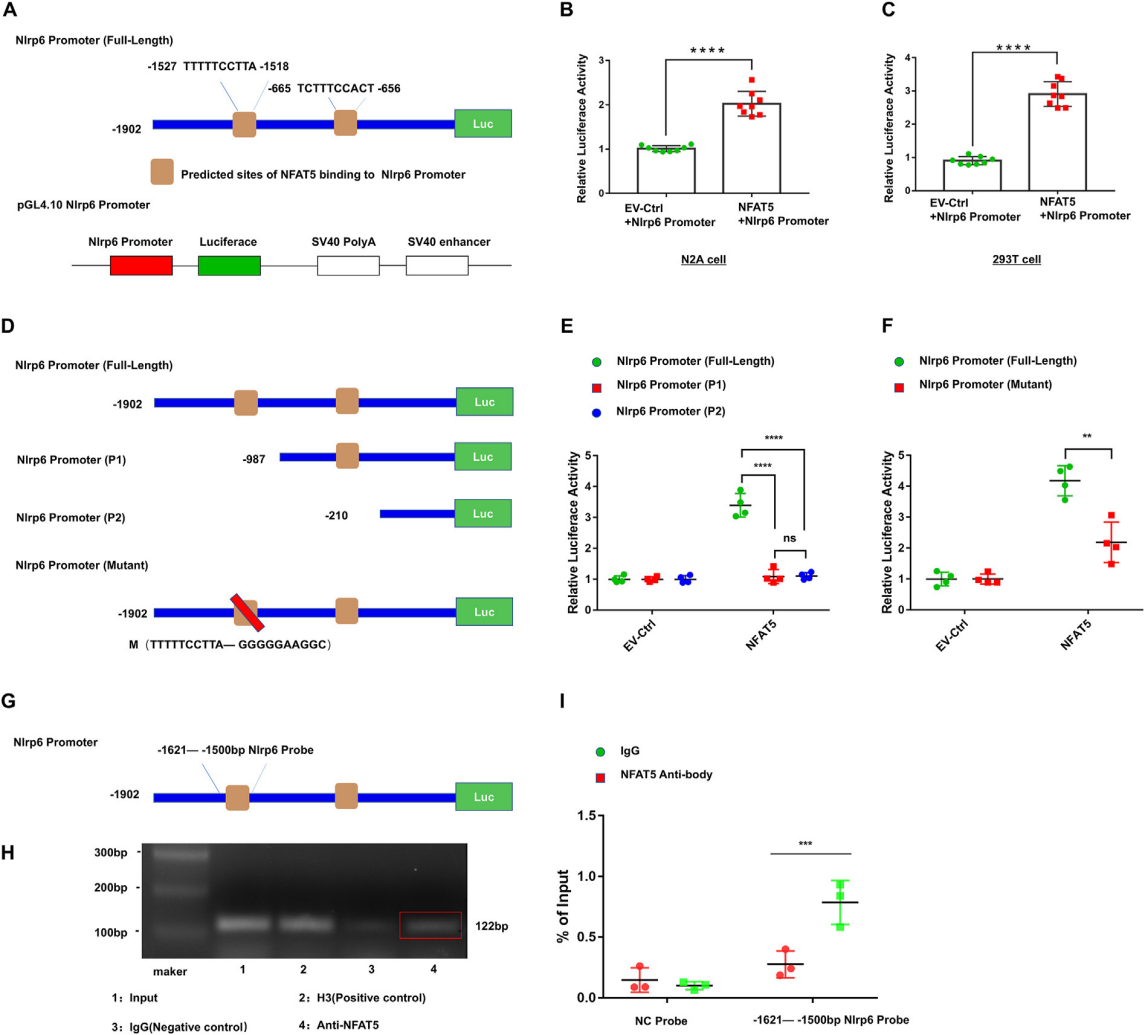
NFAT5 is a transcription factor for the *Nlrp6* promoter. **(A)** The diagram illustrating the predicted binding sites of NFAT5 on the *Nlrp6* promoter. **(B, C)** Dual luciferase reporter assay of pGL4.10-*Nlrp6* promoter after co-transfection with NFAT5 overexpressing (NFAT5) or control vector (TR) in N2A and 293T cell lines (*n* = 8). **(D)** Construction of two *Nlrp6* promoter fragments (P1 and P2) and a mutant of the *Nlrp6* promoter. **(E)** Dual luciferase reporter assay of pGL4.10-*Nlrp6* promoter (full length) or pGL4.10-*Nlrp6* promoter fragments after co-transfection with NFAT5 overexpressing (NFAT5) or control vector (TR) into 293T cell line (*n* = 4). **(F)** Dual luciferase reporter assay of pGL4.10-*Nlrp6* promoter (full length) or pGL4.10-*Nlrp6* promoter mutant after co-transfection with NFAT5-overexpressing (NFAT5) or control vector (TR) into 293T cell line (*n* = 4). **(G)** The schematic depicting the positions of the *Nlrp6* promoter probe for chromatin immunoprecipitation-quantitative PCR/PCR. **(H, I)** Chromatin immunoprecipitation with NFAT5 antibody in BV2 cells was analyzed by PCR (*n* = 3) (H) and quantitative PCR (*n* = 3) (I). H3 represents the positive control group, and IgG represents the negative control. The data were presented as mean with standard deviation. ∗∗*p* < 0.01, ∗∗∗*p* < 0.001, and ∗∗∗∗*p* < 0.0001; ns, no statistical significance.

NFAT5, a transcription factor, has been reported to regulate the downstream genes via interacting with the gene promoter elements.^30^ Our study aimed to determine whether NFAT5 interacted with the *Nlrp6* promoter element. To achieve this, we utilized the Jasper2020 online tool to predict potential regions where NFAT5 could bind to the *Nlrp6* promoter. Our analysis displayed two potential NFAT5-binding sites (−1527 bp to −1518 bp and −665 bp to −656 bp) on the mouse *Nlrp6* promoter (Fig. 7A). To investigate the role of NFAT5 on the transcriptional activity of the *Nlrp6* promoter, we cloned the full-length (FL) of the mouse *Nlrp6* promoter into pGL4.10 (a luciferase reporter plasmid). Subsequently, the mouse NFAT5 plasmid (pLenti6-Nfat5-Flag) or an empty vector plasmid (EV-Ctrl), together with the pGL4.10 *Nlrp6* promoter and the reference plasmid pGL4.74 (rLuc), were co-transfected into both human HEK 293T cells and mouse Neuro-2a cell line (N2A) cells for 48 h. Compared with the EV-Ctrl group, the relative luciferase activity of the *Nlrp6* promoter was notably amplified in the NFAT5 group, indicating that NFAT5 enhances the transcriptional activity of the *Nlrp6* promoter (Fig. 7B, C). Next, we fragmented the *Nlrp6* promoter into fragment 1 (P1) and fragment 2 (P2) (Fig. 7D). The results exhibited that both fragment 1 (P1) and fragment 2 (P2) exhibited markedly decentralized luciferase activity, compared with the *Nlrp6* promoter (FL) group (Fig. 7E, F). Subsequently, we mutated thymine (T) to guanine (G) and mutated pyrimidine (C) to adenosine (A) in the −1527 bp to −1518 bp region on the wild-type *Nlrp6* promoter, forming a *Nlrp6* mRNA mutant (Fig. 7D). Further dual-luciferase results demonstrated *Nlrp6* promoter mutant displayed significantly reduced luciferase activity, compared with the *Nlrp6* promoter (wild-type) group, suggesting that NFAT5 may promote the activity of the *Nlrp6* promoter via the sequence −1527 bp to −1518 bp of *Nlrp6* promoter. Moreover, we designed a *Nlrp6* PCR probe targeting the −1527 bp to −1518 bp region of the *Nlrp6* promoter and conducted ChIP-PCR and ChIP-quantitative PCR assays to investigate the interaction between NFAT5 and the *Nlrp6* promoter (Fig. 7G). The ChIP-PCR results indicated that *Nlrp6* promoter fragments could be amplificated in the anti-NFAT5 group and the amplificated fragments were around 122 bp. However, the amplification of *Nlrp6* promoter fragments in the IgG group was not observed (Fig. 7H). Additionally, the result of ChIP-quantitative PCR illustrated that the amplification of *Nlrp6* promoter fragments was significantly higher in the anti-NFAT5 group than in the IgG group, which revealed that NFAT5 protein could interact with the *Nlrp6* promoter (Fig. 7I). Take in together, our results indicated that NFAT5 interacted with *Nlrp6* promoter, and NFAT5 promoted NLRP6 transcriptional activity via *Nlrp6* promoter −1527 bp to −1518 bp element.

### NFAT5 regulates NLRP6 mRNA stability through the *Nlrp6* 5′UTR

**Figure 8:**
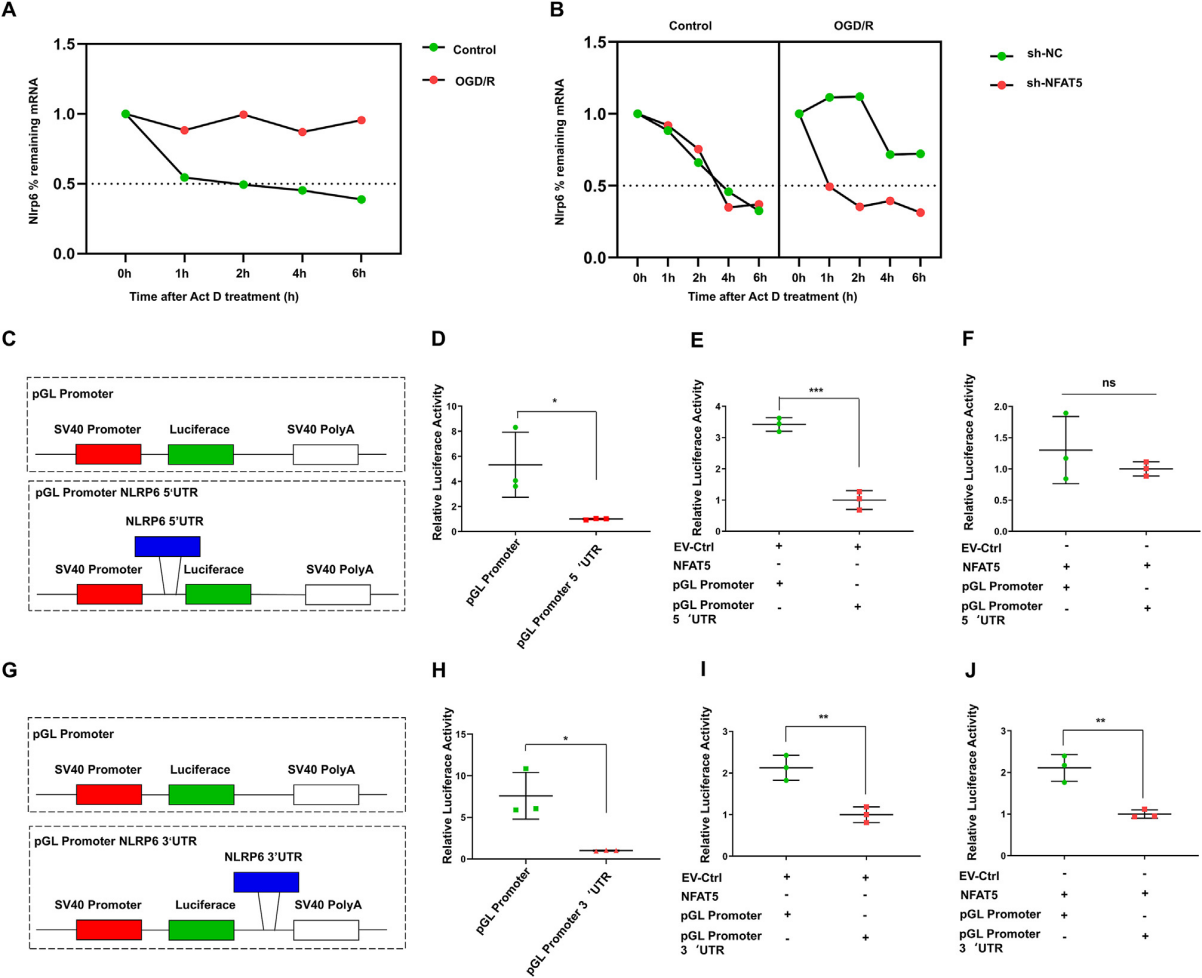
NFAT5 regulates NLRP6 mRNA stability through the *Nlrp6* 5′UTR. **(A)** Relative expression of *Nlrp6* mRNA in wild-type BV2 cell line after OGD/R modeling and actinomycin D (Act D) treatment for 0, 1, 2, 4, and 6 h (*n* = 3). (**B**) Relative expression of *Nlrp6* mRNA in sh-NC and sh-NFAT5 BV2 cell lines after OGD/R modeling and Act D treatment for 0, 1, 2, 4, and 6 h (*n* = 3). (**C**) Construction of pGL-promoter *Nlrp6* 5′UTR plasmid containing the mouse *Nlrp6* 5′UTR region for dual-luciferase reporter assay. **(D**–**F)** Relative luciferase activity of pGL-promoter or pGL-promoter *Nlrp6* 5′UTR after co-transfection with NFAT5-overexpressing (NFAT5) or control vector (TR) into the 293T cell line. Relative luciferase activity was determined and normalized to Renilla reference luciferase activity (*n* = 3). **(G)** Construction of pGL-promoter *Nlrp6* 3′UTR plasmid containing the mouse *Nlrp6* 3′-UTR region for dual-luciferase reporter assay. **(H**–**J)** Relative luciferase activity of pGL-promoter or pGL-promoter *Nlrp6* 3′UTR after co-transfection with NFAT5 overexpressing (NFAT5) or control vector (TR) into the 293T cell line (*n* = 3). The relative luciferase activity was determined and normalized to Renilla reference luciferase activity. The data were presented as mean with standard deviation. ∗*p* < 0.05, ∗∗*p* < 0.01, and ∗∗∗*p* < 0.001; ns, no statistical significance.

We proceeded to investigate whether NFAT5 played a role in regulating the stability of *Nlrp6* mRNA. To address this question, we administered actinomycin D to inhibit mRNA synthesis in both control and OGD/R-treated BV2 cells. We then assessed NLRP6 mRNA half-life through quantitative PCR analysis. Our findings revealed that in the control group, the half-life of *Nlrp6* mRNA was approximately 2 h, whereas it extended to 6 h after OGD/R treatment (Fig. 8A). To further explore the impact of NFAT5 on NLRP6 mRNA stability, we treated BV2 cells with NFAT5 knockdown with actinomycin D under OGD/R conditions. As shown in Figure 8B, silencing NFAT5 reduced the half-life of NLRP6 mRNA. Given that mRNA stability is associated with both the 3′UTR and 5′UTR regions of genes,^31^^,^^32^ we cloned the mouse *Nlrp6* 5′UTR and mouse *Nlrp6* 3′UTR into the pGL4.10-SV40 promoter to generate the pGL4.10-*Nlrp6* 5′UTR and pGL4.10-*Nlrp6* 3′UTR plasmids, respectively. Subsequently, we conducted dual-luciferase reporter assays (Fig. 8C, G). The results showed that both pGL4.10-*Nlrp6* 3′UTR and pGL4.10-*Nlrp6* 5′UTR led to a decrease in luciferase activity (Fig. 8D, H), indicating that both the 3′UTR and the 5′UTR of NLRP6 negatively impact the stability of NLRP6 mRNA. To delve deeper into the effect of NFAT5 on *Nlrp6* 5′UTR and *Nlrp6* 3′UTR, we co-transfected either the control vector (EV-Ctrl) or the NFAT5 plasmid along with the pGL4.10-SV40 promoter-*Nlrp6* 5′UTR or pGL4.10-SV40 promoter-*Nlrp6* 3′UTR plasmid into 293T cells. The EV-Ctrl was unable to rescue the reduced dual-luciferase activity induced by *Nlrp6* 3′UTR and *Nlrp6* 5′UTR (Fig. 8E, I). However, NFAT5 significantly restored dual-luciferase activity attenuated by *Nlrp6* 5′UTR but not by *Nlrp6* 3′UTR (Fig. 8F, J). In summary, our results indicate that NFAT5 regulates NLRP6 mRNA stability in BV2 cells following OGD/R treatment, with NFAT5 potentially maintaining the stability of NLRP6 mRNA through the *Nlrp6* 5′UTR.

## Discussion

**Figure 9:**
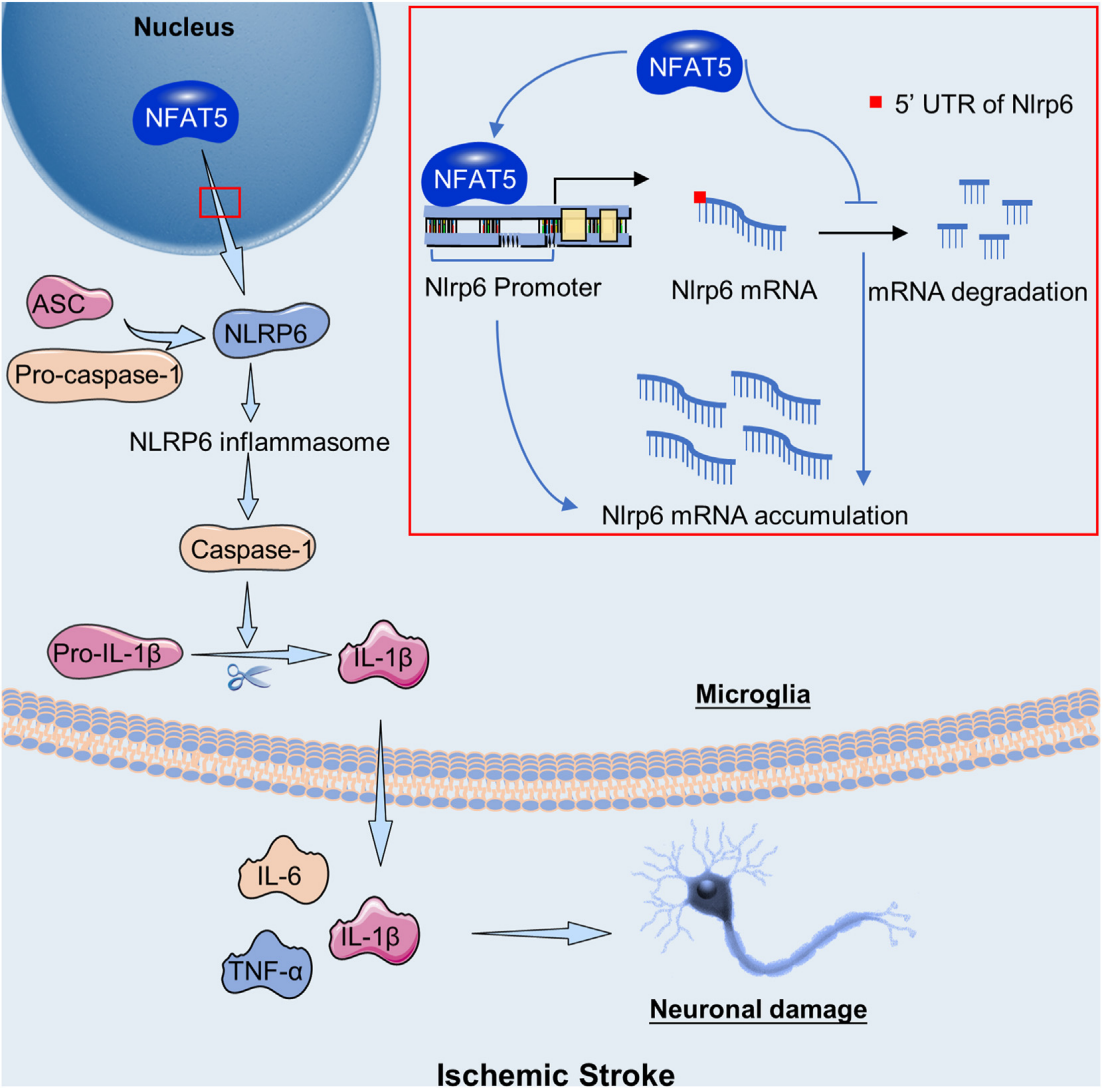
The schematic of microglial NFAT5 aggravating neuroinflammation and neuronal injury via mediating NLRP6 inflammasome following ischemic stroke.

In this study, we confirmed the role of microglial NFAT5 in the acute phase of ischemic stroke and provided insights into the underlying mechanism of NFAT5 in microglia-induced neuroinflammation. Firstly, our findings demonstrated that MACO modeling and OGD/R modeling induced enhanced expression of NFAT5. Secondly, we found that the suppression of microglial NFAT5 ameliorated cerebral tissue injury in the MCAO model. Thirdly, we observed that microglial NFAT5 augmented the synthesis of pro-inflammatory molecules, stimulated microglial activation, facilitated the infiltration of neutrophils, and ultimately triggered neuronal apoptosis. Fourthly, NFAT5 was identified as a regulator of NLRP6 inflammasome activation, as well as NLRP6 mRNA and protein levels. Moreover, NFAT5 was shown to enhance the transcriptional activity of the *Nlrp6* promoter through the −1527 bp to −1518 bp region of the *Nlrp6* promoter. Lastly, but notably, our results indicated that NFAT5 played a role in regulating the mRNA stability of *Nlrp6* through the 5′UTR of *Nlrp6* (Fig. 9).

Following ischemic stroke, the ischemic core area exhibits irreversible infarction, whereas the ischemic penumbra, surrounding the ischemic core area, forms a reversible damage characterized by neuronal apoptosis.^33^ The excessive and robust inflammatory response exacerbates brain injury in the ischemic penumbra, leading to irreversible damage and expansion of the cerebral infarction.^34^ NFAT5, a new member of the Rel family of transcription factors, has recently been reported to be involved in inflammatory response.^35^ Previous research has indicated NFAT5 expression and response to MCAO in astrocytes,^36^ neurons,^37^ and brain endothelial cells.^38^ Kunze et al have demonstrated that MCAO induces endothelial NFAT5 expression; endothelial NFAT5 deficiency impairs reperfusion capacity after ischemic stroke, exacerbates neuronal injury, and worsens post-ischemic functional deficits.^38^ In this work, our results showed that suppression of microglial NFAT5 attenuated neuroinflammation, ameliorated neuronal apoptosis around the penumbra region, and reduced the extent of cerebral infarction. Therefore, NFAT5 may mediate neuronal apoptosis and the extent of cerebral infarction via regulating neuroinflammation around the penumbra region. In contrast to our results, a previous study demonstrated that the heterozygous (NFAT5^+/−^) mice exhibited more pronounced cerebral edema and larger cerebral infarction after hypoxia/ischemia compared with wild-type mice.^39^ We postulated that the differences in the outcome of the two studies may be due to the different *in vivo* models. In our MCAO model, the middle cerebral artery was occluded for 1 h, while in the aforementioned study, the hypoxia/ischemia was maintained for up to 2 h. However, there are a number of previous studies that are in agreement with our findings. Jeong et al have observed that microglial NFAT5 deficiency hinders neuronal death and lipopolysaccharide-induced memory impairment in the context of lipopolysaccharide-induced neuroinflammation modeling.^40^ In addition, heterozygous (NFAT5^+/−^) mice have been documented to attenuate hippocampal inflammation in high-fat diet-induced diabetes compared with wild-type mice.^41^ Moreover, it has been reported that NFAT5 deficiency moderates the inflammatory pain 42. Consistently, we found that microglial NFAT5 silencing impeded microglial activation and neutrophil infiltration in the MCAO model. Taken together, our current experimental data suggested the microglial NFAT5 may exacerbate neuronal damage and neurological deficits via promoting neuroinflammation in MCAO modeling.

Activated microglia serve as a pivotal factor in initiating neuroinflammation following ischemic stroke.^43^ To distinguish the activated microglia from blood-derived monocytes/macrophages remains challenging due to their striking morphological and functional similarities. However, the utilization of GFP bone marrow chimeric mice contributes to the distinction between these two cell types. Investigators discovered that resident microglia (GFP-negative) were activated in significant numbers and infiltrated the infarcted region within 24 h following ischemic stroke, whereas macrophage (GFP-positive) infiltration was not observed within three days after cerebral ischemic stroke.^44^ Subsequently, a significant number of macrophages (GFP-positive) infiltrated the peri-infarct area of brain tissue between days three and seven after ischemic stroke.^45^ Therefore, in our present work, we hypothesized that during the first 24 h after cerebral ischemia/reperfusion, the infarcted region of brain tissue is primarily infiltrated by activated microglia rather than macrophages.

Increased proinflammatory factors from microglia have been identified as a critical role in the pathology of ischemic stroke. Excess IL-1β and TNF-α have been reported to modulate synaptic plasticity and cause glutamate excitotoxicity, dopaminergic cell loss, progressive neurodegeneration, and motor disabilities.^46^^,^^47^ In addition, IL-1β and TNF-α have been reported to induce the expression of IL-6 and some chemokines, which further promote neutrophil infiltration and amplify neuroinflammation.^48^ IL-1β and IL-1β antibodies have been reported to reduce cerebral edema and cerebral infarction in the mouse MCAO model.^49^^,^^50^ Similarly, TNF-α antibody has also been documented to reduce cerebral infarct volume in MCAO mice.^51^ Therefore, inhibiting the proinflammatory factors IL-1β and TNF-α may be a potential treatment for MCAO-induced neuroinflammation and neuronal injury. Histological studies have provided evidence that NFAT5 plays a role in promoting the expression of proinflammatory factors. Li et al found that knockdown of NFAT5 in alveolar macrophages could markedly reduce the levels of TNF-α and IL-1β.^52^ In addition, Jeong’s study explained that the knockdown of NFAT5 in microglial BV2 cells could result in a reduction of lipopolysaccharide-induced TNF-α and cyclooxygenase-2 (COX-2) expression.^40^ Consistently, our results displayed that knockdown of NFAT5 decreased the expression of IL-1β, TNF-α, and IL-6, minimized the number of neutrophils and microglia, and alleviated neuronal injury in the MCAO and OGD/R models. In general, we speculated that microglial NFAT5 may exacerbate neuroinflammation and neuronal injury by facilitating the expression of pro-inflammatory factors.

Our previous study discovered that NLRP6 aggravated neuroinflammation and brain injury after MCAO modeling.^20^ NLRP6, a novel member of the NLR family, is responsible for recruiting pro-caspase-1 to form NLRP6 inflammasome by recruiting ASC. Hara et al have reported that suppression of NLRP6 leads to a reduction in caspase-1 cleavage and IL-1β secretion.^29^ In our present work, we demonstrated that NFAT5 could regulate the mRNA and protein levels of NLRP6, thereby modulating the activation of NLRP6 inflammasome. Moreover, our study discovered that NFAT5 interacted with the *Nlrp6* promoter region and promoted transcriptional activity of the *Nlrp6* promoter. Similar to our study, Ma et al also observed that NFAT5 could interact with the *Nlrp3* promoter, resulting in NLRP3 inflammasome activation and the subsequent development of atherogenesis.^53^ Notably, NLRP6 and NLRP3, both belonging to the NLR family, share similarities in their DNA sequences.^19^ Consequently, it is plausible that NFAT5 may interact with the promoters of both Nlrp3 and *Nlrp6*, thereby modulating the activity of both the NLRP3 inflammasome and the NLRP6 inflammasome. Several studies have confirmed that NLRP3 inflammasome activation facilitates the development of MCAO-induced brain injury.^54^^,^^55^ However, based on the current evidence, we could not conclude whether NLRP6 inflammasome or NLRP3 inflammasome was more essential in MCAO-induced neuroinflammation. Despite the focus on post-transcriptional modification of the NLRP6 protein 56, our study sheds light on the regulation of the NLRP6 mRNA. Numerous regions approximately 1000 bp upstream of the NLRP6 transcription start site have been discovered to be associated with transcription factor peroxisome proliferator-activated receptor γ (PPAR-γ).^57^ However, these authors did not provide a detailed explanation of the molecular mechanism underlying the relationship between PPAR-γ and the *Nlrp6* promoter. In this work, we truncated and mutated the *Nlrp6* promoter to investigate the specific region through which NFAT5 affects the activity of the *Nlrp6* promoter. Our results showed that NFAT5, a transcription factor, could promote the transcriptional activity of the *Nlrp6* promoter through the −1527 bp to −1518 bp region of the *Nlrp6* promoter. Recently, our published work has revealed that NPAS4 plays a role in the transcriptional regulation of NLRP6 and influences neuronal focal death during the acute phase of cerebral hemorrhage.^25^ Importantly, our current research has demonstrated that NFAT5, a member of the Rel transcription factor family, not only enhances the transcriptional activity of the *Nlrp6* promoter but also contributes to the stability of the *Nlrp6* mRNA. A previous study documented that the long non-coding RNA TINCR facilitated pyroptosis by stabilizing NLRP3 mRNA.^58^ However, the stability of *Nlrp6* mRNA has not been reported in previous studies. Our study is the first to discover that *Nlrp6* mRNA exhibits increased stability following OGD/R modeling, and both the 5′UTR and 3′UTR of *Nlrp6* could regulate *Nlrp6* mRNA stability. Furthermore, our results revealed that NFAT5 may influence the stability of *Nlrp6* mRNA through the 5′UTR of *Nlrp6*.

Notably, there are certain limitations to our current work. One limitation is that the microglia in our study are not primary cells. Transfection of viruses or siRNAs into primary microglia is prohibitive, making it challenging to knock down microglial NFAT5. Another limitation is that we cannot perform the replication experiments in *in vivo* or *in vitro* models due to the large molecular weight of the NFAT5 protein. Mouse NFAT5 cannot be packaged into AAV viruses or lentiviruses overexpressing NFAT5 for replication experiments. This issue will be addressed in our follow-up study.

## Conclusions

In summary, our data suggested the role of NFAT5 in microglia-induced neuroinflammation both *in vitro* and *in vivo*. It is observed in our study that the knockdown of NFAT5 in microglia decreased the expression of IL-1β, TNF-α, and IL-6, as well as minimized the number of neutrophils and microglia after MCAO modeling. In addition, suppression of NFAT5 in microglia mitigated neuronal loss, reduced the extent of cerebral infarction, and improved limb grip strength in mice after MCAO modeling. Moreover, our results indicate that NFAT5 has the potential to activate the NLRP6 inflammasome by increasing the transcriptional activity of the *Nlrp6* promoter and stabilizing *Nlrp6* mRNA. Our study provides a novel perspective to elucidate the upstream activation mechanism of the NLRP6 inflammasome. Furthermore, we propose that targeting NFAT5 may be a promising approach to prevent microglia-induced neuroinflammation and neuronal apoptosis following ischemic stroke.

## CRediT authorship contribution statement

**Hui Gan:** Writing – review & editing, Writing – original draft, Software, Methodology, Investigation, Funding acquisition, Data curation, Conceptualization. **Mi Zhang:** Writing – original draft, Software, Methodology, Data curation. **Yuhao Duan:** Validation. **Ailiyaer Palahati:** Investigation. **Qi He:** Funding acquisition. **Junyi Tan:** Validation. **Yong Li:** Project administration. **Xuan Zhai:** Funding acquisition. **Jing Zhao:** Writing – review & editing, Supervision, Funding acquisition.

## Ethics declaration

The study was approved by the Ethics Committee of Chongqing Medical University (Chongqing, China). All animal experiments were in strict accordance with the UK’s Animals (Scientific Procedures) Act 1986 and associated guidelines (NIH Publication No. 85-23, revised 1996).

## Data availability

The data used and analyzed in this work are available from the corresponding author on reasonable request.

## Funding

This research was supported by the 10.13039/501100001809National Natural Science Foundation of China (No. 82071305, 82301512, 82302474), 10.13039/100012546Chongqing Postdoctoral Science Foundation (China) (No. cst2023NSCQ-BHX0121), Program for Youth Innovation in Future Medicine (Chongqing Medical University, China), and Chongqing Talent Plan “Contract Program” (China) (No. cstc2022ycjh-bgzxm0057).

## Conflict of interests

Jing Zhao is an editor for *Genes & Diseases* and was not involved in the editorial review or the decision to publish this article. All authors declared no other competing interests.
