# Supplementary material for: Microglial NFAT5 aggravates neuroinflammation via mediating NLRP6 inflammasome in experimental ischemic stroke
Source: Genes Dis. 2025 Apr 1;12(6):101614. doi: 10.1016/j.gendis.2025.101614 (PMC12359169; doi:10.1016/j.gendis.2025.101614)
Supplement: Multimedia component 1 [file mmc1.doc]

**Supplementary Material**
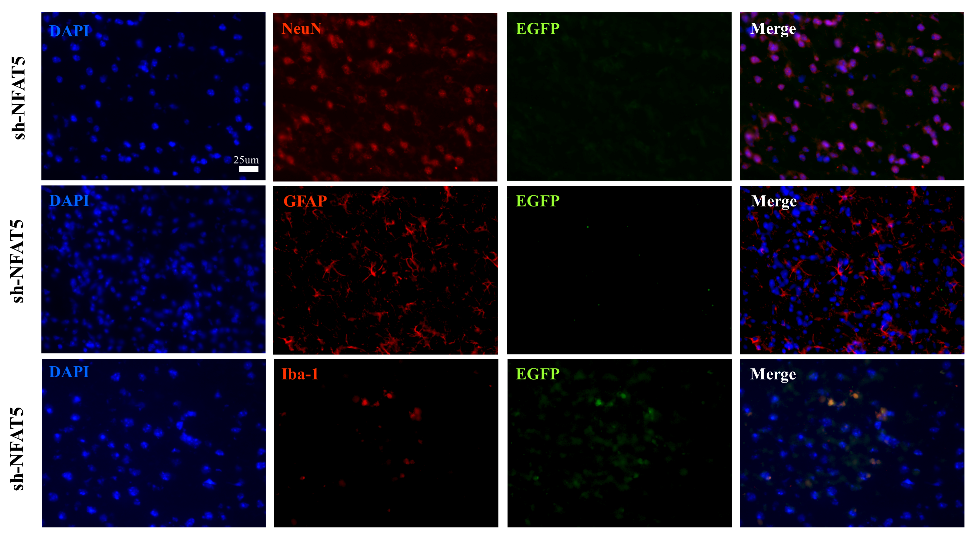


**Figure** **Supplementary 1. Characterization of rAAV-specific expression in microglia.** Immunofluorescence was used to verify the specific expression of rAAV in microglia. The enhanced green fluorescent protein (EGFP) was encoded by rAAV. In addition, Neurons were stained red with NeuN, astrocytes were stained red with GFPA, and microglia were stained red with Iba-1. Scale bar, 25 μm.


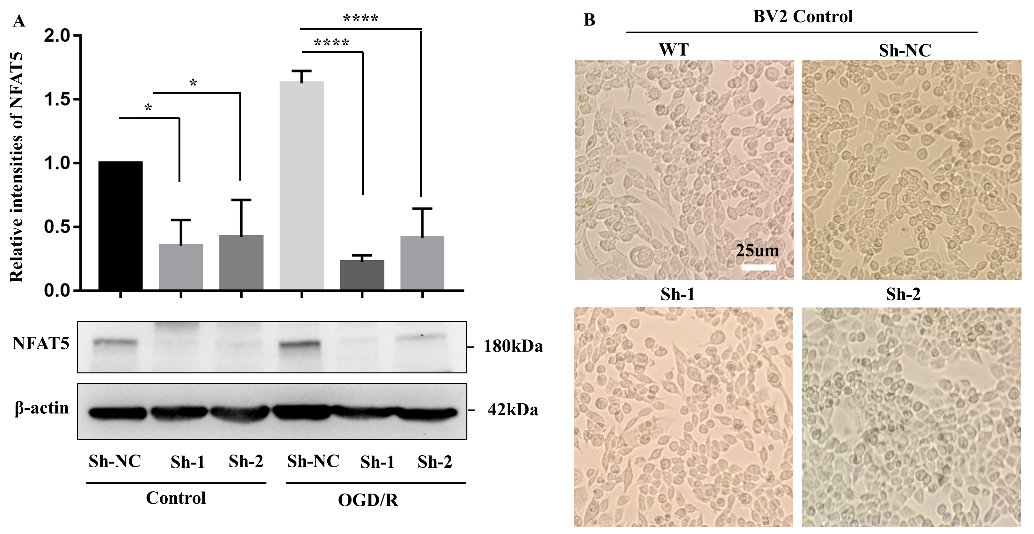


**Figure Supplementary 2. Screening of optimal BV2 cell lines for stable knockdown of NFAT5.** (A) Western blot was used to screen out the optimum BV2 cell lines stably knocking down NFAT5 and relative protein level was normalized to β-actin. The data are shown as mean ± SD.*p<0.05； ****p<0.0001；n=3. (B) The cellular morphology of BV2 cells. Scale bar, 25μm.
